# Correlation between autoimmune thyroid disease and vitiligo: An analytical study

**DOI:** 10.6026/973206300213800

**Published:** 2025-10-31

**Authors:** Sahitya Bandari, Mohammed Zakiullah Shareef, E. Emi Goldy, Naresh Vishwanath Iyer Murali, R. Keerthi Vardhini, Shanmukha Koppolu

**Affiliations:** 1 Department of Emergency Medicine, Royal Balckburn teaching Hospital, East Lancashire hospitals NHS trust, Blackburn, United Kingdom; 2 Department of Internal medicine, Satyadev hospital, Andhra Pradesh, India; 3 Department of Biochemistry, Panimalar Medical College Hospital and Research Institute Varadharajapuram, Poonamallee, Chennai, Tamil Nadu, India; 4 Department of General Medicine, Dr Br Ambedkar Medical College, Bangalore, Karnataka, India; 5 Department of Dermatology Venereology and Leprosy, Madha Medical College and Research Institute, Kovur, Chennai-600128, Tamil Nadu, India; 6 Department of Trauma and Orthopaedics, Barts NHS Trust, London, England/ UK

**Keywords:** Vitiligo, autoimmune thyroid disease, anti TPO, anti TG, thyroid autoimmunity

## Abstract

The association between autoimmune thyroid disease (AITD) and vitiligo in adults is of interest. Anti TPO and anti TG antibody levels were measured in vitiligo patients and matched controls. Vitiligo subjects had significantly higher thyroid autoantibody prevalence than controls. Thus, we show routine thyroid screening in vitiligo. Further longitudinal studies are warranted.

## Background:

Vitiligo is a chronic autoimmune skin condition characterized by loss of pigmentation. It affects an estimated 0.5-2% of the worldwide population [1]. The etiology of vitiligo is complex and multifactorial, involving immune-mediated destruction of melanocytes, oxidative stress and genetic vulnerability and predisposition [2]. Within the various reported comorbidities of vitiligo, autoimmune thyroid disease (AITD) disorders- such as Hashimoto's thyroiditis and Graves' disease- were highlighted to be among the most commonly reported [3]. The association between AITD and vitiligo is well supported by epidemiological cohort studies. In one of the largest meta-analyses of 37 studies with a total of 78,714 vitiligo patients, vitiligo was shown to have significantly higher odds of AITD (OR ≈ 5.88), anti-thyroid peroxidase (anti-TPO) antibody positivity (OR ≈ 3.84), and anti-thyroglobulin (anti-TG) antibody positivity (OR ≈ 3.51) [4]. Another recent pooled analysis of 48 studies estimated that approximately 14.3% of patients with vitiligo had coexisting AITD conditions [5]. Subclinical hypothyroidism in vitiligo has been demonstrated to be the most frequently reported thyroid disorder, occurring in nearly 6% of vitiligo patients [6]. Other case-control studies have demonstrated that vitiligo patients have consistently greater serum titers of both anti-TPO and anti-TG antibodies compared to healthy controls providing further evidence for the immunopathological overlap between vitiligo and AITD conditions [7]. Together, the findings from these studies should raise awareness for thyroid screening as part of routine practice among patients with vitiligo, particularly mindful of the high prevalence of asymptomatic disease [8]. Therefore, it is of interest to report the prevalence and spectrum of thyroid dysfunction and autoantibody positivity among patients with vitiligo in a clinical cohort.

## Materials and Methods:

This case-control study recruited 50 vitiligo patients and 50 age- and sex-matched healthy controls from Metro Medical College between January and December 2024. Clinical vitiligo diagnosis was confirmed by dermatology. Exclusion criteria included known thyroid disease or other autoimmune conditions. All participants underwent serum anti TPO and anti TG antibody testing via ELISA and thyroid function (TSH, FT3 and FT4) was assessed. Statistical analyses compared means and proportions using t-tests and chi-square tests, with significance set at p < 0.05.

## Results:

The study examined 50 patients with vitiligo and 50 healthy controls that were matched for sex and age to explore the association with autoimmune thyroid disease. The mean age of the cohort of patients with vitiligo was 32.4 ± 11.8 years and did not differ from controls, and 60% of both study groups were female. Serum antibody testing indicated that the mean anti-TPO levels were significantly higher in patients with vitiligo vs. controls (212.3 ± 266.1 vs. 12.4 ± 30.5 IU/mL, p < 0.001). Additionally, mean anti-TG was significantly elevated in patients with vitiligo (75.2 ± 112.5 vs. 3.1 ± 6.3 IU/mL, p < 0.001). Approximately 50% of patients with vitiligo had positive anti-thyroid autoantibodies; whereas, only 4% of controls tested positive. Thyroid autoimmunity tends to be significantly positively associated with vitiligo. Furthermore, subclinical hypothyroidism was detected in 16% of patients with vitiligo while no controls demonstrated thyroid dysregulation. The odds ratio suggested that patients with vitiligo tend appear to have greater risk of developing thyroid autoimmunity compared to patient controls. Furthermore, prevalence of antibody positivity was greater in females and patients with early-onset vitiligo. This data supports the prospect of screening thyroid autoimmunity in patients with vitiligo, particularly in female patients and younger patients. Finally, collectively these data suggest an immunologic overlap in patients with vitiligo and autoimmunity to the thyroid which further emphasize the need monitoring on a greater clinical scale.

Table 1 (see PDF) compares the baseline characteristics, showing similar age and gender distribution between vitiligo patients and controls, ensuring demographic comparability. Table 2 (see PDF) presents serum antibody levels, indicating significantly higher anti-TPO and anti-TG levels among vitiligo patients compared to controls. Table 3 (see PDF) describes antibody positivity and thyroid status, showing that half of the vitiligo patients were antibody-positive and 16% had subclinical hypothyroidism, while controls had minimal involvement. Table 4 (see PDF) summarizes association measures, revealing that vitiligo patients had a markedly increased risk of thyroid autoimmunity, with statistically significant results. Table 5 (see PDF) compares gender-wise antibody positivity, demonstrating higher positivity rates among female vitiligo patients. Table 6 (see PDF) interprets the relationship between age at vitiligo onset and antibody positivity, showing that earlier onset was associated with higher antibody prevalence.

## Discussion:

This research establishes a clear link between vitiligo and thyroid autoimmunity, finding a positivity rate of anti-TPO and/or anti-TG antibodies of 50% among vitiligo patients and a significantly smaller fraction of healthy controls. These results support past findings that individuals with vitiligo are at increased risk of developing autoimmune thyroid disease and are compatible with larger studies suggesting a greater odd of thyroid autoantibodies in this population [9]. Additionally, the presence of subclinical hypothyroidism in this cohort underscores the underlying autoimmune processes driving both vitiligo and thyroid disease. While clinically overt thyroid disease was rare, the relatively high rate of subclinical thyroid disease suggests that thyroid autoimmunity can remain asymptomatic in individuals with vitiligo for long period of time before becoming clinically apparent. This too supports the idea that thyroid autoimmunity represents a continuum of autoimmune activity that starts with autoantibody formation and can eventually transition into clinically significant abnormalities in endocrine function [10, 11]. The occurrence of vitiligo alongside autoimmune thyroid disease is also well known in the autosomal polyglandular syndrome's context, particularly in type 3c, in which vitiligo and thyroid autoimmunity frequently coexist. The immunopathogenetic mechanism for this association is likely multifactorial, potentially including cross-reactivity of shared autoantigens, altered cytokine response, and a common genetic predisposition that affects melanocytes in addition to thyroid tissue. Female sex, a family history of autoimmunity, and longer duration of vitiligo have consistently been associated with higher prevalence of thyroid autoantibodies, and similar aspects were noted in this cohort [12, 13]. From a clinical standpoint, these data suggest that routine thyroid monitoring in patients with vitiligo is important, even in the absence of symptoms or abnormal thyroid function studies. Early detection of subclinical thyroid disease may afford a timely opportunity for surveillance and intervention that could prevent the progression to overt hypothyroidism and decrease adverse health effects associated with compromised thyroid function, particularly in high-risk populations such as women and patients with a strong family history of autoimmune disease [14, 15].

Despite these strengths, it is important to acknowledge some limitations. The moderate sample size may reduce the potential generalizability of the findings and the cross-sectional design does not allow the authors to determine the temporal or causal relationship between vitiligo and thyroid autoimmunity. Exploring this further with longitudinal perspectives, and repeated measurements of thyroid function and antibodies, would help to characterize the natural history of thyroid involvement in this condition and enhance the strength of the evidence for causality. Although there is a strong association between vitiligo and thyroid autoimmunity, it is prudent to pursue thyroid screening as a part of standard care for people with vitiligo. Including routine antibody and thyroid function testing in clinical care pathways for routine evaluation may increase the likelihood of early detection with the potential for patient-centered management options for thyroid conditions. Evaluating the transition from subclinical to overt thyroid dysfunction, and determining the appropriateness of retesting protocols and the frequency for future follow-up as part of guideline recommendations are both warranted with studies moving forward.

## Conclusion:

Vitiligo has a strong link to autoimmune thyroid disease and is characterized by a higher prevalence of thyroid autoantibodies and subclinical hypothyroidism. Routine screening for thyroid function and thyroid autoantibodies in patients with vitiligo may allow for early identification and management of any abnormalities. This may serve to limit complications and improve shoulder outcomes in such patients.
